# Circulating tumor necrosis factor receptors are associated with mortality and disease severity in COVID-19 patients

**DOI:** 10.1371/journal.pone.0275745

**Published:** 2022-10-11

**Authors:** Tomohito Gohda, Maki Murakoshi, Yusuke Suzuki, Makoto Hiki, Toshio Naito, Kazuhisa Takahashi, Yoko Tabe

**Affiliations:** 1 Department of Nephrology, Juntendo University Faculty of Medicine, Tokyo, Japan; 2 Department of Emergency Medicine, Juntendo University Faculty of Medicine, Tokyo, Japan; 3 Department of Cardiovascular Biology and Medicine, Juntendo University Graduate School of Medicine, Tokyo, Japan; 4 Department of Research Support Utilizing Bioresource Bank, Juntendo University Graduate School of Medicine, Tokyo, Japan; 5 Department of General Medicine, Juntendo University Faculty of Medicine, Tokyo, Japan; 6 Department of Respiratory Medicine, Juntendo University Faculty of Medicine and Graduate School of Medicine, Tokyo, Japan; 7 Department of Clinical Laboratory Medicine, Juntendo University Faculty of Medicine, Tokyo, Japan; Stanford University School of Medicine, UNITED STATES

## Abstract

**Background:**

Although hyperinflammatory response influences the severity of coronavirus disease 2019 (COVID-19), little has been reported about the utility of tumor necrosis factor (TNF)-related biomarkers in reflecting the prognosis. We examined whether TNF receptors (TNFRs: TNFR1, TNFR2) and progranulin (PGRN) levels, in addition to interleukin 6 (IL-6) and C-reactive protein (CRP), are associated with mortality or disease severity in COVID-19 patients.

**Methods:**

This retrospective study was conducted at Juntendo University Hospital. Eighty hospitalized patients with various severities of COVID-19 were enrolled. Furthermore, serum levels of TNF-related biomarkers were measured using enzyme-linked immunosorbent assay.

**Results:**

Twenty-five patients died during hospitalization, and 55 were discharged. The median (25th and 75th percentiles) age of the study patients was 70 (61–76) years, 44 (55.0%) patients were males, and 26 (32.5%) patients had chronic kidney disease (CKD). When comparing with patients who received and did not receive treatment at the intensive care unit (ICU), the former had a higher tendency of being male and have diabetes, hypertension, and CKD; had higher levels of white blood cells, D-dimer, and lactate dehydrogenase; and had lower body mass index, estimated glomerular filtration rate, and lymphocyte counts. Significant differences were observed in TNFR, PGRN, IL-6, and CRP levels between each severity (mild–severe) group. Furthermore, the serum levels of TNFR, IL-6, and CRP, but not PGRN, in ICU patients were significantly higher than in the patients who were not admitted to the ICU. Multivariate logistic regression analysis demonstrated that high levels of TNFR2 were only associated with mortality in patients with COVID-19 even after adjustment for relevant clinical parameters.

**Conclusions:**

High TNFR2 level might be helpful for predicting mortality or disease severity in patients with COVID-19.

## 1. Introduction

Since December 2019, coronavirus disease 2019 (COVID-19), a severe respiratory illness caused by severe acute respiratory syndrome coronavirus-2 (SARS-Cov-2), has resulted in a pandemic and many deaths. However, most patients have an asymptomatic or a mild illness course [1, 2]. It has been widely known that the hyperinflammatory reaction caused by SARS-CoV-2 infection, called a cytokine storm, may lead to vascular endothelial cell injury and thrombus formation in the blood vessels of each organ, particularly in severe COVID-19 patients, resulting in lung damage, acute respiratory distress syndrome, and multiple organ dysfunction [3]. Various hematological abnormalities have reportedly been associated with the severity of COVID-19 [4]. Rahman et al. reported that levels of C-reactive protein (CRP) in addition to ferritin and d-dimer are good indicators of COVID-19 severity [5].

The global prevalence of diabetes and chronic kidney disease (CKD) is estimated to be around 10% [6, 7]. Both diseases are associated with a marked increase in mortality and risk factors for COVID-19 [8, 9]. Inflammation is closely involved in the pathogenesis of both diseases [10, 11]. Numerous studies have reported that inflammatory biomarkers predict the progression of diabetic kidney disease (DKD) and mortality [12, 13]. We have reported that tumor necrosis factor (TNF)-related biomarkers, such as TNF receptors (TNFR1 and TNFR2) and progranulin (PGRN), are associated with the pathogenesis of obesity, diabetes, and CKD [14–16], and predict GFR loss and/or mortality in patients with diabetes and/or CKD [17–20].

However, the data on the circulating levels of TNF-related biomarkers in COVID-19 patients are limited [21–24]. Therefore, in this study, we evaluated whether the circulating levels of TNFR1, TNFR2, and PGRN, in addition to interleukin 6 (IL-6) and CRP, are associated with disease severity and mortality in 80 hospitalized COVID-19 patients.

## 2. Materials and methods

### 2.1 Patients

This retrospective study was conducted at the Juntendo University Hospital, a 1,051-bed university-affiliated hospital in Tokyo, Japan. We enrolled some hospitalized patients with polymerase chain reaction-confirmed COVID-19 between April 21, 2020 and September 25, 2021. Patients were divided into four categories according to the COVID-19 severity classification, developed by the Ministry of Health, Labor, and Welfare of Japan (S1 Table) [25]. First, we selected 40 patients admitted to the intensive care unit (ICU) and then contemporary 40 patients with less severe illness (non-ICU) from each category [mild (n = 18), moderate I (n = 12), moderate II (n = 10)]. Blood samples were obtained from patients as part of the standard medical hospital procedure on the first day or following admission and frozen at −80°C until assayed. This study was conducted following the tenets of the Helsinki Declaration and was approved by the institutional review board (IRB) of Juntendo University Hospital, Tokyo, Japan (IRB # 20–036). The need for informed consent from individual patients was waived as all samples were de-identified according to the Declaration of Helsinki.

### 2.2 Measurement of serum TNFR1, TNFR2, and PGRN

The serum concentrations of TNFR1, TNFR2, and PGRN were measured using enzyme-linked immunosorbent assay (ELISA) kits (cat. # DRT100, DRT200, DPGRN0; R&D Systems, Minneapolis, MN, USA) as previously described [26, 27]. Serum levels of IL-6 were analyzed using a commercial chemiluminescent enzyme immunoassay (SRL, Tokyo, Japan). The serum CRP was measured through nephelometry, a latex-enhanced immunoturbidimetric assay (QUALIGENT CRP; Sekisui Medical Co., Ltd., Tokyo, Japan) at our institution.

### 2.3 Statistical analyses

Data are expressed as the median (25^th^ and 75th percentiles) or percentage. Cross-sectional comparisons were examined using the Mann–Whitney U, Kruskal–Wallis, chi-squared, and Fisher’s exact tests. Spearman’s correlation coefficient was used to test the correlations between different variables. Univariate and multivariate logistic regression analyses were performed to explore the risk factors associated with mortality. The area under the receiver operating characteristic (ROC) curve was used to evaluate prognostic efficacy. Statistical analyses were performed using SAS software v.9.4 (SAS Institute, Cary, NC, USA). *P*-values of <0.05 were considered statistically significant.

## 3. Results

### 3.1 Clinical characteristics of the patients

Clinical characteristics related to disease severity and survival are shown in Table 1 and S2 Table, respectively. The median (25th and 75th percentiles) age was 70 (61–76) years, and 44 (55.0%) patients were male.

**Table 1:** Clinical characteristics and inflammatory markers related to disease severity at the time of admission.

Characteristics	Non-ICU (n = 40)	ICU (n = 40)	p-value
Age (years)	69 (61–75)	72 (61–79)	0.63
Male (%)	26 (27.3%)	32 (72.7%)	<0.0001
BMI	28.4 (26.4–29.7)	25.4 (23.9–27.5)	0.005
Systolic BP (mmHg)	119 (111–134)	115 (103–126)	0.32
Diastolic BP (mmHg)	72 (65–88)	70 (61–77)	0.29
Hypertension (%)	9 (22.5%)	18 (45.0%)	0.03
Diabetes (%)	5 (12.5%)	14 (35.0%)	0.02
CKD (GFR <60) (%)	7 (17.5%)	19 (47.5%)	0.004
Prior CVD (%)	4 (10.0%)	9 (22.5%)	0.13
eGFR (ml/min/1.73 m^2^)	76 (65–88)	70 (32–84)	0.005
WBC (/μL)	4300 (3350–6000)	6950 (4400–10450)	<0.0001
Lymphocyte (/μL)	1081 (803–1414)	554 (413–851)	<0.0001
Ferritin (ng/mL)	279 (141–543)	848 (376–1461)	<0.0001
LDH (IU/L)	210 (170–249)	313 (267–436)	<0.0001
D-dimer (μ/mL)	1.6 (1.2–2.1)	2.6 (1.8–5.0)	<0.0001
TNFR1 (pg/mL)	1797 (1546–2623)	3818 (2511–5885)	<0.0001
TNFR2 (pg/mL)	3716 (3019–4936)	6488 (5307–8435)	<0.0001
PGRN (ng/mL)	119 (79–152)	165 (97–201)	0.02
CRP (mg/dL)	0.89 (0.16–3.55)	5.15 (3.53–12.36)	<0.0001
IL-6 (pg/mL)	8.7 (3.4–20.8)	43.3 (18.2–105.3)	<0.0001

Data are presented as mean ± standard deviation, median (quartiles), or %.

Abbreviations: BMI, body mass index; BP, blood pressure; CKD, chronic kidney disease; CRP, C-reactive protein; CVD, cardiovascular disease; eGFR, estimated glomerular filtration rate; ICU, intensive care unit; IL-6, interleukin 6; LDH, lactate dehydrogenase; PGRN, progranulin; TNFR, tumor necrosis factor receptor; WBC, white blood cell.

BMI: non-ICU (n = 40), ICU (n = 37); Ferritin: non-ICU (n = 40), ICU (n = 39); IL-6: non-ICU (n = 38), ICU (n = 36)

As shown in Table 1, patients who received treatment in the ICU had a higher tendency to be male and have diabetes, hypertension, and CKD than those who received treatment in non-ICU wards. Patients who received treatment in the ICU had significantly higher levels of white blood cells (WBCs), D-dimer, and lactate dehydrogenase (LDH); however, they had significantly lower body mass index (BMI), lymphocyte count, and estimated glomerular filtration rate (eGFR) than those who received treatment in the non-ICU wards. In contrast, no difference was found in age, frequency of prior CVD, or systolic/diastolic BP.

### 3.2 Circulating inflammatory marker levels

The levels of all inflammatory markers were significantly higher among patients who received treatment in the ICU than those who did not (Table 1). Table 2 shows significant differences in the inflammatory marker levels among patients with varying disease severity. The levels of CRP and IL-6 were significantly lower among patients with mild disease compared with all other groups, and were significantly higher among patients in the severe group compared with the mild and moderate I groups. However, the levels of these markers were not significantly different between the severe and moderate II groups. No significant difference in TNFR1 or TNFR2 levels was observed between the mild, moderate I, and moderate II groups; however, the severe group exhibited significantly higher levels of these markers than all other groups. Therefore, CRP and IL-6 levels increased stepwise with the disease severity, whereas TNFR levels increased steeply during severe illness.

**Table 2:** Circulating levels of inflammatory markers in relation to disease severity.

	Mild (n = 18)	Moderate I (n = 12)	Moderate II (n = 10)	Severe (n = 40)	P
TNFR1	1647 (1430–1870)	2176 (1686–2623)	2432 (1606–3206)	3818 (2511–5885)*^†^^‡^	<0.0001
TNFR2	3117 (2822–3754)	4017 (3215–5100)	4342 (3319–5127)	6488 (5307–8435)*^†^^‡^	<0.0001
PGRN	103 (70–143)	118 (109–159)	158 (130–181)	165 (97–201)^‡^	0.0009
IL-6	3.4 (1.9–4.8)	17.2 (6.8–23.9)^§^	25.0 (11.5–38.2)^#^	43.3 (18.2–105.3)^†^^‡^	<0.0001
CRP	0.16 (0.07–0.40)	2.33 (0.38–4.88)^§^	3.46 (1.87–4.86)^#^	5.15 (3.53–12.36)^†^^‡^	<0.0001

Abbreviations: CRP, C-reactive protein; IL-6, interleukin 6; PGRN, progranulin; TNFR, tumor necrosis factor receptor.

IL6; Mild: IL-6 (n = 17), Moderate II (n = 9), Severe (n = 36)

Notes:

*Severe vs. Moderate II p < 0.05,

^†^Severe vs. Moderate I p < 0.05,

^‡^Severe vs. Mild p < 0.05;

^§^Moderate I vs. Mild p < 0.05;

^#^Moderate II vs. Mild p < 0.05

### 3.3 Association between TNFRs, PGRN, IL-6, CRP, and clinical parameters

Significant correlations were noted between many of the measured parameters (Table 3). The clinical parameters in routine practice, such as WBCs, lymphocytes, ferritin, D-dimer, and LDH, showed correlation coefficients between 0.30 and 0.67, except for the relationship between WBCs and lymphocytes (r = −0.03). Other inflammatory markers, such as TNFR1, TNFR2, IL-6, and CRP, except for PGRN, were associated with moderate-to-strong correlation coefficients between 0.69 and 0.94. Moreover, TNFR1, TNFR2, IL-6, and CRP, except for PGRN, were moderately associated with all clinical parameters in routine clinical practice (correlation coefficients between 0.42 and 0.67). The relationship between eGFR and inflammatory markers were shown in S3 Table.

**Table 3:** Results of Spearman correlation coefficients in relation to clinical and inflammatory markers.

	**TNFR1**	**TNFR2**	**PGRN**	**IL-6**	**CRP**	**WBC**	**Lymphocyte**	**Ferritin**	**D-dimer**	**LDH**
**TNFR1**		0.94*	0.16	0.69*	0.69*	0.54*	−0.45*	0.45*	0.67*	0.56*
**TNFR2**			0.24^§^	0.72*	0.73*	0.46*	−0.43*	0.51*	0.66*	0.61*
**PGRN**				0.32^‡^	0.24^§^	−0.19	−0.22	0.15	0.01	−0.00
**IL-6**					0.78*	0.42^†^	−0.48*	0.62*	0.63*	0.61*
**CRP**						0.45*	−0.51*	0.62*	0.64*	0.58*
**WBC**							−0.03	0.30^‡^	0.53*	0.48*
**Lymphocyte**								−0.42*	−0.48*	−0.33^‡^
**Ferritin**									0.44*	0.59*
**D-dimer**										0.64*

Abbreviations: BMI, body mass index; BP, blood pressure; CKD, chronic kidney disease; CRP, C-reactive protein; CVD, cardiovascular disease; eGFR, estimated glomerular filtration rate; ICU, intensive care unit; IL-6, interleukin 6; LDH, lactate dehydrogenase; PGRN, progranulin; TNFR, tumor necrosis factor receptor; WBC, white blood cell.

*p < 0.0001,

^†^p < 0.001,

^‡^p < 0.01,

^§^ p < 0.05

IL-6 (n = 74), Ferritin (n = 79), Other markers (n = 80)

### 3.4 Association between inflammatory markers and mortality using univariate and multivariate logistic analyses

**Fig 1:**
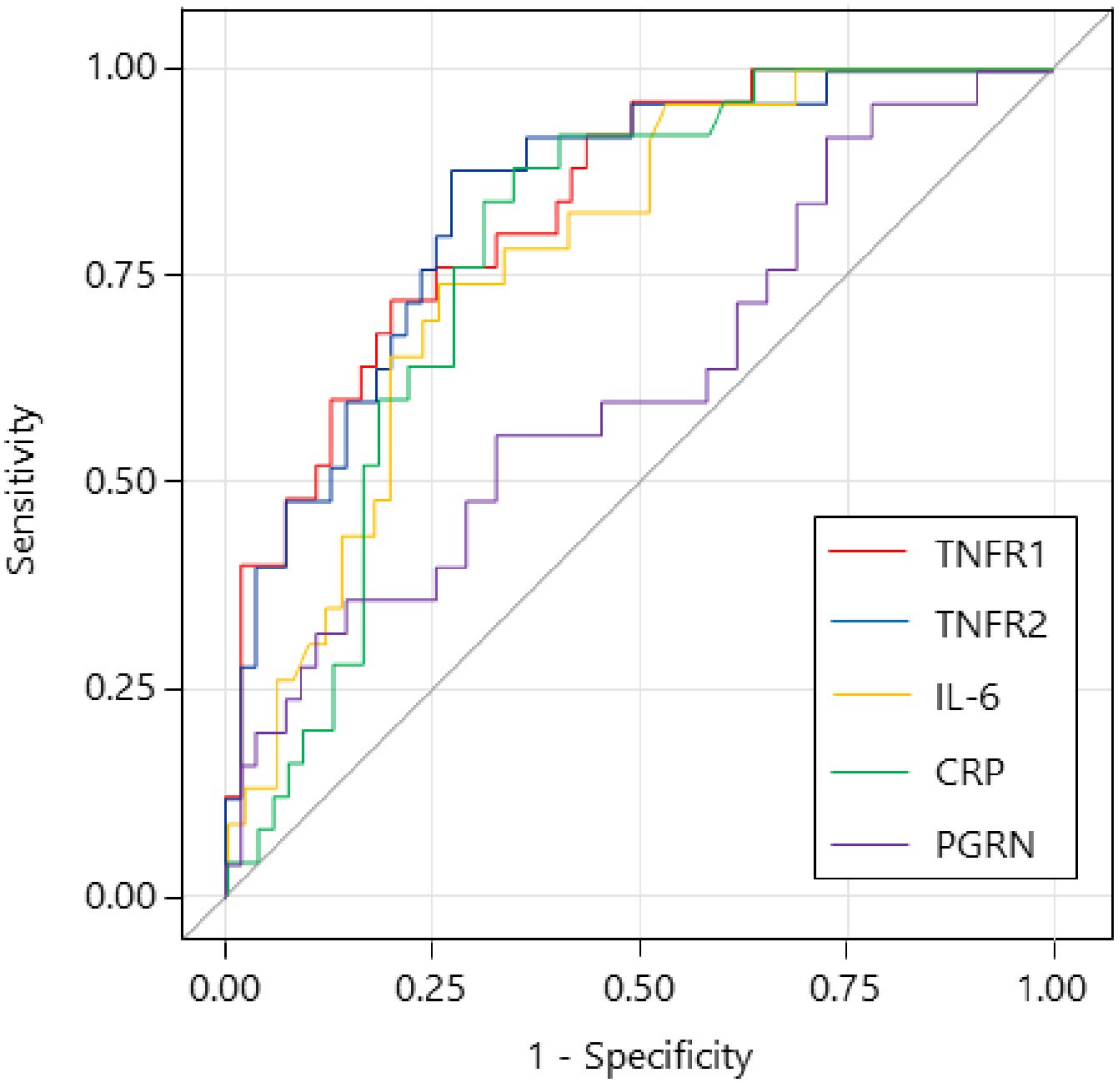
Graph illustrating the diagnostic value of biomarkers for mortality in COVID-19 patients. The diagnostic value of tumor necrosis factor receptor 1 and 2, Interleukin-6, C-reactive protein, and progranulin for mortality. The area under curve values were 0.834, 0.841, 0.778, 0.781, and 0.622, respectively.

As shown in Table 4, many clinical and inflammatory markers except for PGRN were associated with mortality, following univariate logistic regression analysis. The ROC was plotted using levels of inflammatory markers to assess the efficacy of the predictors of mortality (Table 5 and Fig 1). Both TNFR1 and TNFR2 demonstrated favorable diagnostic performance according to ROC analysis (Table 5 and Fig 1); the area under curve (AUC) value for TNFR2 was 0.841 (95% confidence interval [CI] 0.752–0.931) with a cutoff of 5278 pg/mL, 88% sensitivity, and 73% specificity. The AUC value for TNFR1 was 0.834 (95% CI 0.743–0.925) with a cutoff of 3247 pg/mL, 72% sensitivity, and 80% specificity. Both CRP and IL-6 showed fair diagnostic performance according to ROC analysis (Table 5 and Fig 1). Age (odds ratio [OR], 1.19; 95% CI, 1.08–1.31; p = 0.0005), WBCs (OR, 1.88; 95%CI, 1.01–3.51; p = 0.047), and lymphocyte count (OR, 0.16; 95%CI, 0.06–0.44; p = 0.0003) were associated with mortality in the multivariate model using only clinical parameters (clinical model). When each inflammatory marker was added to the clinical model (Table 6), high levels of TNFR2 were only associated with mortality after adjustment for relevant clinical parameters.

**Table 4:** Results of univariate logistic regression analysis of the clinical parameters and inflammatory markers for mortality.

Characteristics	OR (95% CI)	p-value
Clinical parameters		
Age	1.12 (1.05–1.19)	0.0007
Male	2.87 (1.03–7.96)	0.04
BMI	0.96 (0.81–1.13)	0.61
Hypertension	1.92 (0.72–5.11)	0.19
Systolic BP	0.89 (0.67–1.18)	0.43
Diastolic BP	0.50 (0.30–0.83)	0.01
Diabetes	1.39 (0.47–4.11)	0.55
CKD	6.00 (2.13–16.94)	0.0007
Prior CVD	2.17 (0.64–7.28)	0.21
WBC	2.09 (1.23–3.55)	0.007
Lymphocyte	0.37 (0.20–0.67)	0.001
Ferritin	1.51 (0.91–2.50)	0.11
LDH	2.09 (1.25–3.49)	0.005
D-dimer	2.37 (1.33–4.21)	0.003
Inflammatory markers		
TNFR1	4.62 (2.13–10.00)	0.0001
TNFR2	7.69 (3.36–17.62)	0.0001
PGRN	1.63 (0.98–2.73)	0.06
IL-6^§^	3.16 (1.61–6.20)	0.0008
CRP	4.23 (1.81–9.89)	0.0009

^§^IL-6 (n = 74)

Abbreviations: BMI, body mass index; BP, blood pressure; CI, confidence interval; CKD, chronic kidney disease; CRP, C-reactive protein; CVD, cardiovascular disease; eGFR, estimated glomerular filtration rate; ICU, intensive care unit; IL-6, interleukin 6; LDH, lactate dehydrogenase; OR, odds ratio; PGRN, progranulin; TNFR, tumor necrosis factor receptor; WBC, white blood cell.

Data are presented as odds ratio (95% confidence interval). All odds ratios are for a continuous Log10 change in biomarker levels.

**Table 5:** Results of receiver operating characteristic analysis of promising markers for mortality of COVID-19.

Biomarker	AUC	95% CI	Cut off value	Sensitivity	Specificity
TNFR1	0.834	0.743–0.925	3247 pg/mL	72%	80%
TNFR2	0.841	0.752–0.931	5278 pg/mL	88%	73%
PGRN	0.622	0.486–0.758	153 ng/mL	56%	65%
IL-6	0.778	0.671–0.886	28.2 mg/dL	70%	75%
CRP	0.781	0.680–0.882	3.5 pg/mL	88%	65%

AUC, area under the curve; CI, confidence interval; CRP, C-reactive protein; IL-6, interleukin 6; PGRN, progranulin; TNFR, TNF receptor.

**Table 6:** Results of multivariate logistic regression analysis of the inflammatory markers for mortality.

	OR (95% CI)*	p-value
TNFR1	1.12 (0.88–10.62)	0.08
TNFR2	4.17 (1.14–15.24)	0.03
PGRN	1.69 (0.81–3.52)	0.16
IL-6^§^	1.91 (0.69–5.34)	0.21
CRP	1.96 (0.70–5.51)	0.20

*Adjustment for age, white blood cells, and lymphocyte count.

^§^IL-6 (n = 74)

Abbreviations: CI, confidence interval; CRP, C-reactive protein; IL-6, interleukin 6; OR, odds ratio; PGRN, progranulin; TNFR, tumor necrosis factor receptor.

Data are presented as odds ratio (95% confidence interval). All odds ratios are for a continuous Log10 change in biomarker levels.

## 4. Discussion

In the present study, we measured the serum levels of TNF-related biomarkers in various severities of 80 hospitalized COVID-19 patients, of which 25 died during hospitalization. We found that older age, being male, low diastolic BP, lymphocytopenia, high WBC levels, high LDH and D-dimer levels, and high levels of inflammatory markers were related to mortality. Furthermore, the levels of TNFR 1 and 2 in ICU-treated patients (severe illness) were significantly higher than those in non-ICU-treated patients (mild-to-moderate illness), and high levels of TNFR2 were only associated with mortality after adjustment of relevant factors.

The implication of TNFRs and IL-6 in COVID-19 severity appears to be slightly different. IL-6 and CRP levels elevated with the progression of disease severity, whereas TNFR levels steeply increased at the onset of severe illness. Therefore, increase in IL-6 and CRP might be helpful to distinguish the early stage (mild to moderate) of COVID-19 patients. However, these biomarker levels remained unchanged between patients with moderate II and severe illness. The levels of TNFR did not differ between patients with mild-to-moderate illness, although the TNFR levels in patients with severe illness were significantly higher than in those with moderate II illness. These profile differences may also explain why TNFR is a better predictor of mortality than IL-6 in COVID-19 patients.

Haga et al. demonstrated that a disintegrin and metalloprotease 17 (ADAM17) are required for spike-protein-induced shedding of the angiotensin-converting enzyme 2 (ACE2) ectodomain, and increased ADAM17 activity is related to internalization of the spike protein of SARDS-CoV [28]. Exacerbation of ADAM17 activity has been proposed as a possible mechanism underlying the inflammatory immune responses and activation of the coagulation cascade associated with COVID-19 infection [29]. Not only TNFα, IL-6R, and ACE2 but also TNFR1 and TNFR2 were reported to be shed by ADAM17 sheddase [29, 30]. The biological effects of TNF arise through binding to both TNFRs. Palacious *et al*. [22] demonstrated that increased TNFR levels are not due to excessive production, but to increased ADAM17 expression. Notably, TNFR1 levels in COVID-19 patients with severe illness were higher than those with mild illness, but did not differ from those with moderate illness, whereas TNFR2 levels did not differ between each severity group. Unlike their study, both TNFR levels were associated with disease severity, as indicated by the strong correlation (r = 0.94) between TNFR1 and TNFR2 in the present study. These differences may be partially attributed to differences in ethnicities, differences in ELISA kit, and differences in clinical stratification of COVID-19, among other etiologies.

We have previously reported that TNFR and PGRN levels are not only negatively associated with eGFR [26] but also predict eGFR loss and mortality in patients with diabetes and/or CKD [19, 20, 31]. Furthermore, TNF inhibitor, etanercept, improves the progression of DKD by inhibiting the anti-inflammatory action of the TNFα–TNFR2 pathway [14]. However, in this study, the relationship between TNFRs and eGFR was not as strong (TNFR1, r = 0.47; TNFR2, r = 0.44) as in our previous reports in patients with diabetes and CKD [26], indicating that elevated TNFR levels in COVID-19 patients cannot be adequately explained by reduced eGFR alone.

PGRN expressions are upregulated in mouse models of sepsis and involved in host defense against sepsis [32]. Furthermore PGRN binds to TNFR and inhibits inflammatory signals [33]. Yao *et al*. [34] reported that patients with COVID-19 had higher PGRN levels than healthy controls, and that these levels decreased after successful treatment. In the present study, we demonstrated that PGRN levels were higher among ICU-treated patients; however, these values were not predictive of mortality in patients with COVID-19.

This study has several limitations which should be acknowledged. First, selection bias may exist owing to the fact that this study was conducted retrospectively at a single institution and patients were of the same ethnicity. Therefore, it is unknown whether these results can be generalized to other ethnicities. Second, the length from the disease onset to the patients’ admission might be different, which might influence each biomarker level. Third, due to this relatively small number of study patients it was not possible to adjust all relevant risk factors for mortality in multivariate analysis and it does not reflect the global population with COVID-19. Fourth, we did not measure levels of biomarkers at different time points during the period of hospitalization. Therefore, it is unknown whether the levels of these marker changed after recovery from COVID-19. Lastly, there are some missing data for BMI, ferritin, and IL-6.

In conclusion, our findings suggest that inflammatory marker levels may be associated with disease severity. In particular, IL-6 and CRP levels might be useful to distinguish between early-stage (mild to moderate) COVID-19 patients. In contrast, TNFR levels might help distinguish COVID-19 patients who require ICU care. Moreover, high levels of TNFR2 could effectively predict in-hospital mortality independent of relevant clinical parameters in patients with COVID-19.

## Supporting information

S1 Table Severity classification of COVID-19 patients developed by the Ministry of Health, Labor, and Welfare (MHLW) of Japan. (PDF)

S2 Table Clinical characteristics and inflammatory markers related to the mortality. (PDF)

S3 Table Results of Spearman correlation coefficients in relation to estimated glomerular filtration rate and inflammatory markers. (PDF)
