# Supplementary material for: Circulating tumor necrosis factor receptors are associated with mortality and disease severity in COVID-19 patients
Source: PLoS One. 2022 Oct 11;17(10):e0275745. doi: 10.1371/journal.pone.0275745 (PMC9553057; doi:10.1371/journal.pone.0275745)
Supplement: S1 Table — (PDF) [file pone.0275745.s001.pdf]

S1 Table. Severity classification of COVID-19 patients developed by the Ministry of Health, Labor, and Welfare (MHLW) of Japan.

Severity	Oxygen saturation	Symptoms<br>Clinical status
Mild	SpO2 ≥96%	No respiratory symptoms<br>Cough, No dyspnea
Moderate I	93% < SpO2 < 96%	Dyspnea, Pneumonia
Moderate II	SpO2 ≤93%	Requiring oxygen supplementation
Severe		Admission to intensive care unit<br>Requiring mechanical ventilation

SpO2, blood oxygen saturation level
