# Supplementary material for: Circulating tumor necrosis factor receptors are associated with mortality and disease severity in COVID-19 patients
Source: PLoS One. 2022 Oct 11;17(10):e0275745. doi: 10.1371/journal.pone.0275745 (PMC9553057; doi:10.1371/journal.pone.0275745)
Supplement: S2 Table — (PDF) [file pone.0275745.s002.pdf]

S2 Table. Clinical charasteristics and inflammatory markers according to the mortality.

Characteristics	Survivor (n = 55)	Nonsurvivor (n = 25)	p-value
Age (years)	65 (60, 74)	75 (70, 84)	0.0002
Male (%)	26 (47.3%)	18 (72.0%)	0.04
BMI	27.0 (23.9, 29.4)	26.8 (24.9, 28.0)	0.73
Systolic BP (mmHg)	117 (111, 132)	115 (100, 129)	0.52
Diastolic BP (mmHg)	72 (67, 80)	67 (61, 73)	0.007
Hypertension (%)	16 (29.1%)	11 (44.0%)	0.19
Diabetes (%)	12 (21.8%)	7 (28.0%)	0.55
CKD (GFR <60) (%)	11 (20.0%)	15 (60.0%)	0.0004
Prior CVD	5 (9.1%)	7 (28.0)%	0.03
eGFR (ml/min/1.73m^2)	78 (65, 88)	52 (23, 77)	0.006
WBC (/µL)	4500 (3500, 7000)	6900 (4600, 9700)	0.008
Lymphocyte (/µL)	984 (665, 1310)	548 (411, 699)	0.0005
Ferritin (ng/mL)	387 (195, 877)	685 (333, 1011)	0.09
LDH (IU/L)	223 (178, 316)	308 (266, 399)	0.002
D-dimer (µ/mL)	1.6 (1.2, 2.3)	2.6 (2.0, 5.7)	0.0005
TNFR1 (pg/mL)	2034 (1606, 3202)	4298 (3148, 6643)	<0.0001
TNFR2 (pg/mL)	4020 (3167, 5809)	6657 (5906, 9677)	<0.0001
PGRN (ng/mL)	123 (80, 177)	161 (103, 203)	0.09
CRP (mg/dL)	1.87 (0.28, 4.67)	5.62 (4.27, 11.88)	<0.0001
IL-6 (pg/mL)	13.8 (3.9, 28.5)	44.9 (22.1, 123.0)	<0.0001

Data are presented as the mean ± SD, median (quartiles), or %.
BMI, body mass index; BP, blood pressure; CKD, chronic kidney disease; CRP, C-reactive protein; CVD, cardiovascular disease; eGFR, estimated glomerular filtration rate; IL-6, interleukin 6; LDH, lactate dehydrogenase; PGRN, progranulin; TNFR, TNF receptor; WBC, white blood cell.
BMI: survivor (n = 55), non-survivor (n = 22); Ferritin: survivor (n = 55), nonsurvivor (n = 24); IL-6: survivor (n = 51), nonsurvivor (n = 23)
