# Supplementary material for: Circulating tumor necrosis factor receptors are associated with mortality and disease severity in COVID-19 patients
Source: PLoS One. 2022 Oct 11;17(10):e0275745. doi: 10.1371/journal.pone.0275745 (PMC9553057; doi:10.1371/journal.pone.0275745)
Supplement: S3 Table — (PDF) [file pone.0275745.s003.pdf]

S3 Table. Results of Spearman correlation coefficients in relation to estimated glomerular filtration rate and inflammatory markers.

	TNFR1	TNFR2	PGRN	IL-6	CRP
eGFR	−0.47*	−0.44*	−0.32‡	−0.23§	−0.22

Abbreviations: CRP, C-reactive protein;eGFR, estimated glomerular filtration ra

*p < 0.0001, ‡p < 0.01, § p < 0.05

IL-6 (n = 74), Ferritin (n = 79), Other markers (n = 80)
